# An extremely rare case of a high-grade pleomorphic cardiac sarcoma and likely cerebral metastasis in a young patient

**DOI:** 10.3332/ecancer.2016.664

**Published:** 2016-08-15

**Authors:** TG Wilson, P Jenkins, A Hoschtitzky, M McCabe

**Affiliations:** Central Manchester Foundation Trust, Oxford Rd, Manchester M13 9WL, UK

**Keywords:** sarcoma, cardiac tumours, cardiac MRI, leiomyosarcoma

## Abstract

To date, there have been less than a 100 confirmed case reports of primary cardiac malignant fibrous histiocytomas, a rare form of sarcoma. In this report, we discuss the case of a 15-year-old girl who initially presented with a histiocytic cerebral sarcoma that was treated with aggressive resection and chemotherapy. Three years later, the same patient developed increasing shortness of breath and was found to have a high-grade pleomorphic undifferentiated cardiac sarcoma that likely represents the primary tumour from which the cerebral lesion metastasised. This represents an extremely unique case; in 2010, a research group in Germany claimed the very first description of a true cardiac sarcoma with brain metastasis [1]. However, even as far back as 1960, there were three case reports [2] and more extensive sarcoma studies recently have revealed further cases [3]. Nevertheless, there have probably been less than 10 cases in the literature up until this point.

## Introduction

This report highlights the case of a young patient who presented with an extremely rare histiocytoma brain tumour that was treated with surgery and extensive chemotherapy. During follow–up, two years later, however, she was found to have a cardiac sarcoma that likely represented the primary tumour of which the original brain deposit was a metastasis. We will present the literature on histiocytomas and primary cardiac and cerebral sarcomas that are all rare and form conclusions to help assist everyday practice when faced with these tumours.

## Report

Cardiac tumours are rare, studies at autopsy have shown a prevalence of 0.001–0.03% [4], and of these, most cardiac tumours are benign, with myxomas accounting for over 50% [5]. Just 25% of cardiac tumours are malignant, and of these, 80% are sarcomas [6], angiosarcomas tend to affect the right side of the heart and other forms of sarcoma (rhabdomyosarcoma, leiomyosarcoma, liposarcoma, osteosarcoma, fibrosarcoma, and malignant fibrous histiocytoma) tend to affect the left-hand chambers [7].

**Figure 1.**
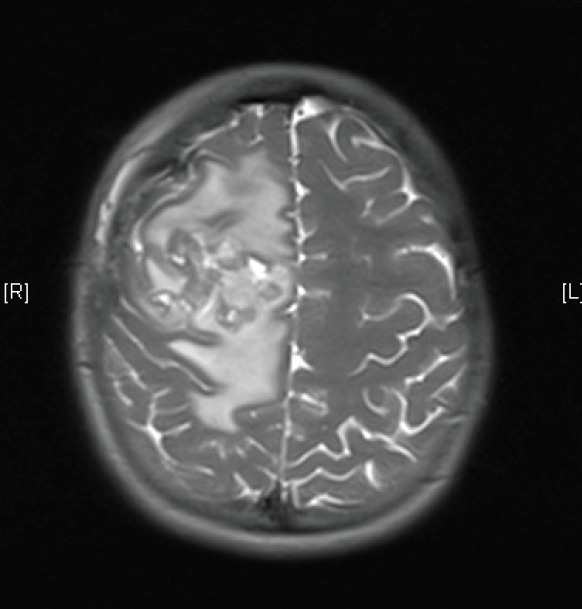
MRI brain 2013.

Our case report starts in April 2013 when a 15-year-old girl presented to her local accident and emergency department with a two-month history of an occipital headache, three weeks of intermittent diplopia and a one-week history of vomiting on multiple occasions daily. An insightful emergency doctor ordered an MRI scan with IV contrast that was performed the same day (Figure 1). This revealed a 5.8 × 4.7 × 4 cm large heterogenous mixed solid and cystic enhancing mass in the right frontal lobe, there was surrounding vasogenic oedema and local mass effect, but no diffusion restriction to indicate tumour infiltration. The on call radiologist felt given the patient’s age and symptoms that this most likely represented a high-grade glioma but also offered differentials of ependymoma or a primitive neuroectodermal tumour (PNET).

The following day, neurosurgeons performed a right-sided frontal-pterional craniotomy to remove the space occupying lesion. Unfortunately, post-operative scans showed significant residual disease and the case was taken to neurosurgical MDT mid-May. Histology, available at this stage, showed lymphocytic infiltration and a high-grade tumour that was INI1, vimentin, and histiocytic markers positive. The likely diagnosis of the brain tumour was suggested to be a histiocytic sarcoma, and further, imaging was suggested to exclude metastases or another primary location. Primary cerebral histiocytomas are even rarer than cardiac histiocytomas and as of 2013, only 10 have ever been reported [8]. Countering this, soft-tissue sarcomas are known very rarely to metastases to the brain, in a study of 3829 patients with soft-tissue sarcomas <1% had cerebral metastases [9] in the study four of these 20 patients were confirmed to be of the histiocytoma subtype [9]. A day after the MDT, a redo right frontal craniotomy was performed by the neurosurgeons to remove the residual tumour.

A CT scan of the patient’s thorax, abdomen, and pelvis was performed to look for other sites of sarcoma, and this was reported as showing no evidence of lesions in either lung, liver, spleen, or kidneys. There was no lymphadenopathy or free fluid in the abdomen and pelvis. The patient was then treated as per UK CCLG High-Grade Glioma guidelines with concomitant focal radiotherapy and temozolomide followed by seven cycles of temozolomide where the patient was able to tolerate 3–4 out of five daily doses in each cycle. The patient concluded treated in March 2014 with no evidence of reoccurrence or any residual disease.

**Figure 2.**
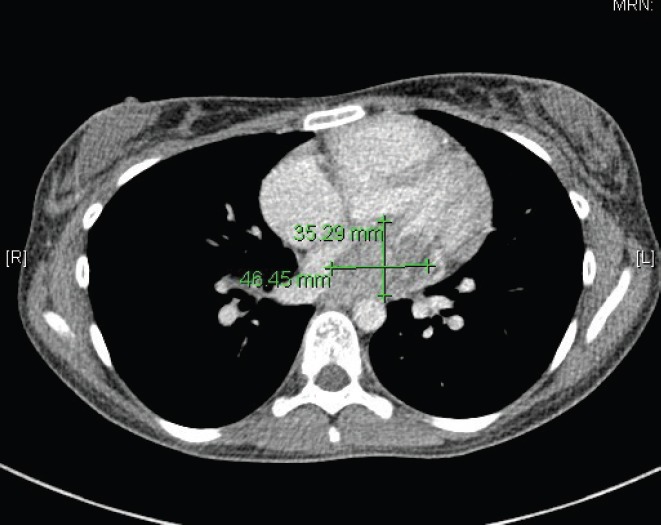
2015 CT thorax with contrast.

**Figure 3.**
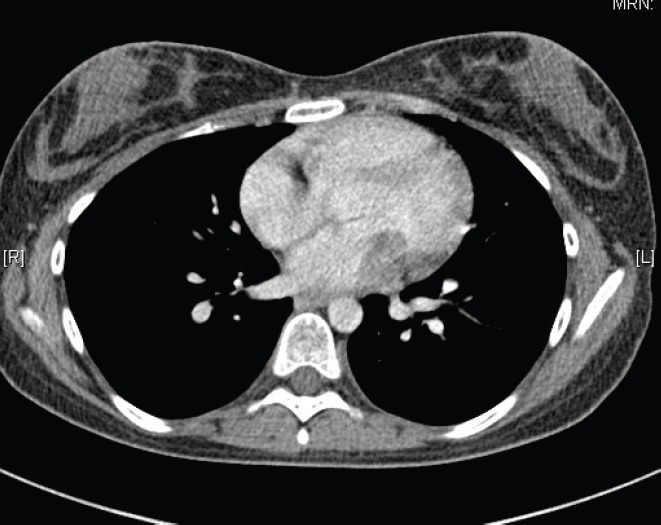
2013 CT thorax with contrast.

In May 2015, the patient now 17 years old presented to her oncology follow-up clinic appointment complaining of increasing shortness of breath over a one-month period combined with lethargy and an isolated anaemia. She was assessed by haematology and diagnosed with Iron deficiency anaemia thought to be secondary to heavy menstruation that was subsequently treated. MRI brain with contrast was performed in mid-June, the frontal lobe appearances were unaltered from the preceding scan, and there was no evidence of disease relapse. However, in August 2015, the patient had a further drop in her haemoglobin this time without iron deficiency and had started having evening and night time fevers. A CT scan was performed to look for a source of the pyrexia of unknown origin. It showed a 4.6 × 3.5 × 3 cm filling defect in the left atrium (Figure 2). The radiologist compared this to the 2013 CT scan and noted that it was present at that time, although only measuring 2 × 2 × 1.6 cm at that time (Figure 3). The mass was centred on the mitral valve and the reporting radiologist suggested this could either represent a chronic vegetation with diseased mitral valve or a mass lesion.

**Figure 4.**
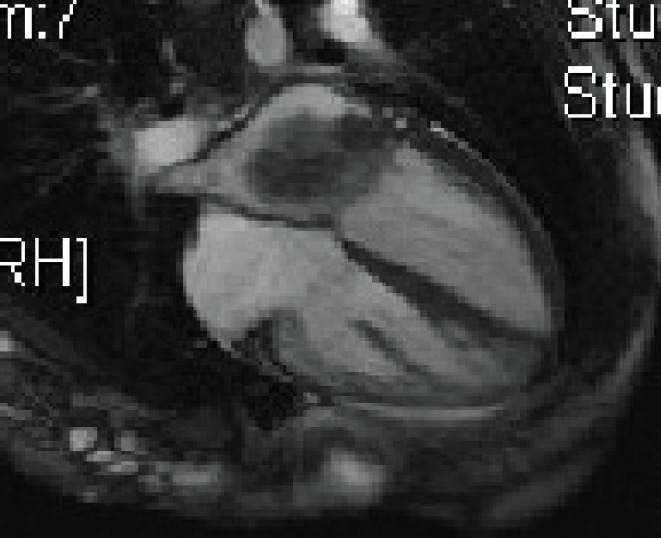
Cardiac MRI 2015.

**Figure 5.**
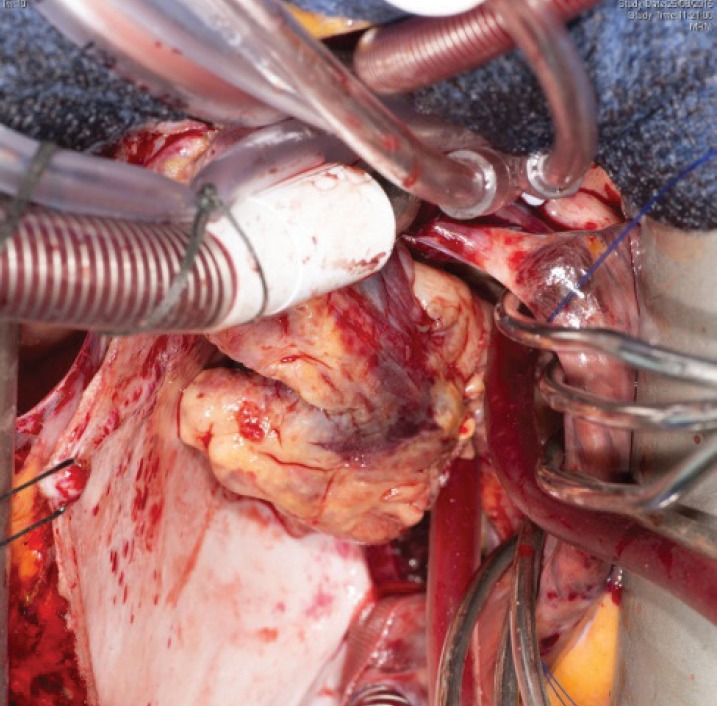
Surgery 2015.

**Figure 6.**
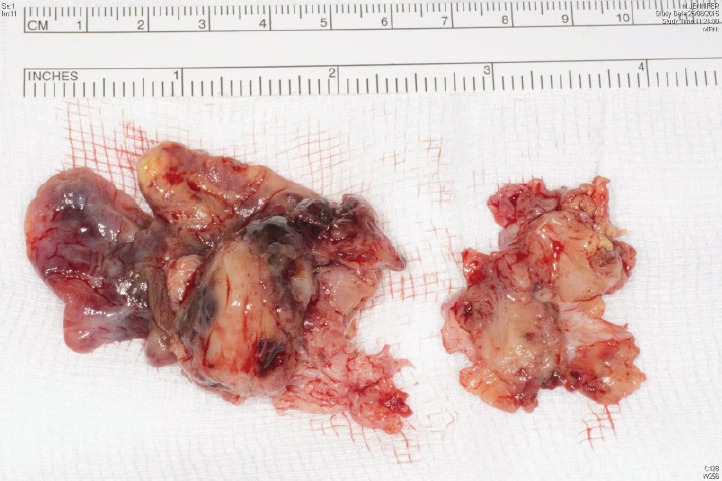
Specimen 2015.

Five days later, a cardiac MRI scan was performed showing a tumour mass attached to the posterior wall of the left atrium lying very close to but not occluding the left lower pulmonary vein (Figure 4). The posterior mitral valve leaflet looked inseparable from the mass. The left ventricular ejection fraction was 55% with a stroke volume of 76 mL and cardiac output of 4.55 L/min. The right ventricular ejection fraction was just 28% with a stroke volume of 33 mL and cardiac output of 3 L/min. The mass was clearly causing obstruction of inflow into the mitral valve and she therefore underwent urgent resection the following morning (Figure 5). The posterior left atrial wall with the pedunculated cardiac tumour was stripped off the endothelial layer of the mitral valve and the defect of the left atrial wall between the pulmonary vein ostia and the posterior mitral valve annulus was reconstructed with a patch of bovine pericardium. A mediastinal lymph node biopsy was also performed and pericardial fluid was sent along with tissue for histology. The 8.5 × 4.5 × 4 cm specimen (Figure 6) was identified as a high-grade pleomorphic undifferentiated sarcoma with MDM2 amplification suggesting that it was an intimal sarcoma. No malignant cells were found in the pericardial fluid. The case including histology was discussed at an Oncology MDT who felt the pleomorphic sarcoma with large atypical cells was on a similar spectrum to the histiocytic sarcoma found two years earlier in the patient’s brain and the loss of the histiocytic marker suggested de-differentiation and unfortunately extremely high levels of mitosis were seen. Some consideration was given to a diagnosis of Li Fraumeni syndrome. Li Fraumeni syndrome is an autosomal dominant condition with very high penetrance characterised by a predisposition to breast cancer, brain tumours, leukaemia, adrenocortical carcinoma, and sarcomas [10]. Li Fraumeni is associated with germline mutations in p53 [11]. The patient was tested and was TP53 negative, and therefore, her case did not represent Li Fraumeni syndrome and it was mostly likely that the cardiac tumour was the original primary tumour and the cerebral lesion represented a metastasis. Two years later, when the cardiac tumour was discovered, the sarcoma had undergone de-differentiation.

Post-operatively, there was an initial deterioration in the patient’s ejection fraction from a pre-operative 51% down to 33%; however, later, in the month, this had an increased back to 48% all values measured by Simpson’s method. A post-operative cardiac MRI did not show any signs of post-operative re-occurrence. The patient has now begun doxycycline/ifosfamide chemotherapy and has had four cycles up until this point.

A Sarcoma centre published 13-year data in 2007; of 1429 patients with sarcoma 14 were primary cardiacsarcomas and only a single one had a cerebral metastasis [12]. Aggressive resection remains the principle management of cardiac sarcomas with improvements in symptoms and mortality, although prognosis remains extremely poor [12]. Case studies of undifferentiated intimal sarcomas in large systemic blood vessels suggest an average prognosis of 11 months [13], although in this case treatment options are more restricted. Case studies looking at primary cardiac sarcomas prognosis suggests life-expectancy is between 9.6 and 16.5 months even with extensive resection and aggressive treatment with chemotherapy and radiotherapy [14].

## Conclusion

In conclusion, this case, albeit of a rare presentation of sarcoma, highlights the need for extensive investigation at presentation, especially if an unusual primary location has been found. PET imaging at the time of diagnosis or after initial surgery would be extremely useful in these cases to assist in the identification of other metastatic sites or primary tumour location that may be missed on plain CT imaging. We would also recommend consideration of cardiac MRI imaging, if there is any potential concern that a primary heart tumour may be involved.
